# Feasibility and accuracy of wearable electrocardiogram monitoring in adult congenital heart disease

**DOI:** 10.1093/europace/euaf247

**Published:** 2025-10-08

**Authors:** Florine Runge, Jonas Brügger, Francisco Javier Ruperti-Repilado, Behnam Subin, Fabian Jordan, Corinne Isenegger, Christian Sticherling, Michael Kühne, Felix Mahfoud, Daniel Tobler, Patrick Badertscher

**Affiliations:** Department of Cardiology, University Hospital Basel, Petersgraben 4, Basel 4031, Switzerland; Cardiovascular Research Institute Basel, University Hospital Basel, Petersgraben 4, Basel 4031, Switzerland; Department of Cardiology, University Hospital Basel, Petersgraben 4, Basel 4031, Switzerland; Cardiovascular Research Institute Basel, University Hospital Basel, Petersgraben 4, Basel 4031, Switzerland; Department of Cardiology, University Hospital Basel, Petersgraben 4, Basel 4031, Switzerland; Cardiovascular Research Institute Basel, University Hospital Basel, Petersgraben 4, Basel 4031, Switzerland; Department of Cardiology, University Hospital Basel, Petersgraben 4, Basel 4031, Switzerland; Cardiovascular Research Institute Basel, University Hospital Basel, Petersgraben 4, Basel 4031, Switzerland; Department of Cardiology, University Hospital Basel, Petersgraben 4, Basel 4031, Switzerland; Cardiovascular Research Institute Basel, University Hospital Basel, Petersgraben 4, Basel 4031, Switzerland; Department of Cardiology, University Hospital Basel, Petersgraben 4, Basel 4031, Switzerland; Cardiovascular Research Institute Basel, University Hospital Basel, Petersgraben 4, Basel 4031, Switzerland; Department of Cardiology, University Hospital Basel, Petersgraben 4, Basel 4031, Switzerland; Cardiovascular Research Institute Basel, University Hospital Basel, Petersgraben 4, Basel 4031, Switzerland; Department of Cardiology, University Hospital Basel, Petersgraben 4, Basel 4031, Switzerland; Cardiovascular Research Institute Basel, University Hospital Basel, Petersgraben 4, Basel 4031, Switzerland; Department of Cardiology, University Hospital Basel, Petersgraben 4, Basel 4031, Switzerland; Cardiovascular Research Institute Basel, University Hospital Basel, Petersgraben 4, Basel 4031, Switzerland; Department of Cardiology, University Hospital Basel, Petersgraben 4, Basel 4031, Switzerland; Cardiovascular Research Institute Basel, University Hospital Basel, Petersgraben 4, Basel 4031, Switzerland; Department of Cardiology, University Hospital Basel, Petersgraben 4, Basel 4031, Switzerland; Cardiovascular Research Institute Basel, University Hospital Basel, Petersgraben 4, Basel 4031, Switzerland

**Keywords:** Smart device, Smartwatch, ACHD, Inconclusive

Wearable devices are increasingly used in clinical practice, particularly for cardiac rhythm monitoring.^1^ Adult congenital heart disease (ACHD) patients are at elevated risk for both atrial and ventricular arrhythmias, contributing significantly to morbidity and mortality.^2,3^ Wearable-based rhythm monitoring may support early arrhythmia detection and improved management in this vulnerable population. We therefore conducted a pilot study to evaluate the feasibility and diagnostic performance of smart devices for arrhythmia detection in ACHD patients.

Adult patients referred to the ACHD clinic at a Swiss tertiary care centre were prospectively enrolled in this prospective, single-centre study. Participants were instructed to record a 30 s electrocardiogram (ECG) three times per week using the KardiaMobile device (AliveCor Inc., Mountain View, CA, USA). Electrocardiograms, recorded at a speed of 25 mm/s, were saved as PDF files and submitted via an online platform at the Basel Wearable Clinic (www.wearableclinic.ch). Rhythm classification was performed both automatically [as sinus rhythm (SR), atrial fibrillation (AF), tachycardia (T), or inconclusive (IC)] and manually by experienced reviewers (J.B. and P.B.) blinded to device output. P-wave analysis was conducted using EP Calipers 3 (EP Studios Inc.), measuring the duration and amplitude of three consecutive P-waves. Results were compared to a control cohort from the Basel Wearable Study (NCT04809922).^4^ The study was approved by the local ethics committee and adhered to the declaration of Helsinki. Continuous variables were reported as medians with interquartile ranges (IQRs), while categorical variables were presented as counts and percentages. The Wilcoxon rank-sum test was applied for comparisons of continuous variables and Fisher’s exact test for categorical variables.

Nine patients (median age 33 years, 44% female) were enrolled. Underlying congenital heart defects included transposition of the great arteries after atrial switch (*n* = 2) and after arterial switch (*n* = 1), congenital corrected transposition of the great arteries (*n* = 1), Fontan circulation (*n* = 1), repaired tetralogy of Fallot (*n* = 1), ventricular septal defect with pulmonary atresia (*n* = 1), repaired aortic coarctation (*n* = 1), and non-corrected cyanotic heart disease (mitral atresia *n* = 1) (*Figure 1*). A total of 133 ECGs were collected [median 11 per patient (IQR 10–27); range 6–31]. One patient did not submit any recordings. Device-based rhythm classification identified 62 ECGs (46.6%) as SR, 4 (3.0%) as AF, 8 (6.0%) as tachycardia, and 59 (44.4%) as IC. Manual review established a rhythm diagnosis in all cases, confirming 102 ECGs as SR and 31 as ectopic atrial arrhythmias. The device’s sensitivity and specificity for detecting sinus rhythm were 52% and 45%, respectively. P-wave analysis was feasible in 102 ECGs; 31 ECGs were excluded due to absent or indistinct P-waves. No significant differences in P-wave characteristics were observed between ECGs classified as SR and IC by the device [duration: 69 ms (56–82) vs. 71 ms (61–86), *P* = 0.362; amplitude: 0.09 mV (0.07–0.13) vs. 0.10 mV (0.09–0.11), *P* = 0.113]. Compared with a control cohort of 90 patients (median age 69 years, 30% female), ACHD patients had significantly shorter P-wave durations [66 ms (57–79) vs. 107 ms (85–124), *P* < 0.001] and lower amplitudes [0.09 mV (0.07–0.11) vs. 0.80 mV (0.67–0.99), *P* < 0.001].

**Figure 1:**
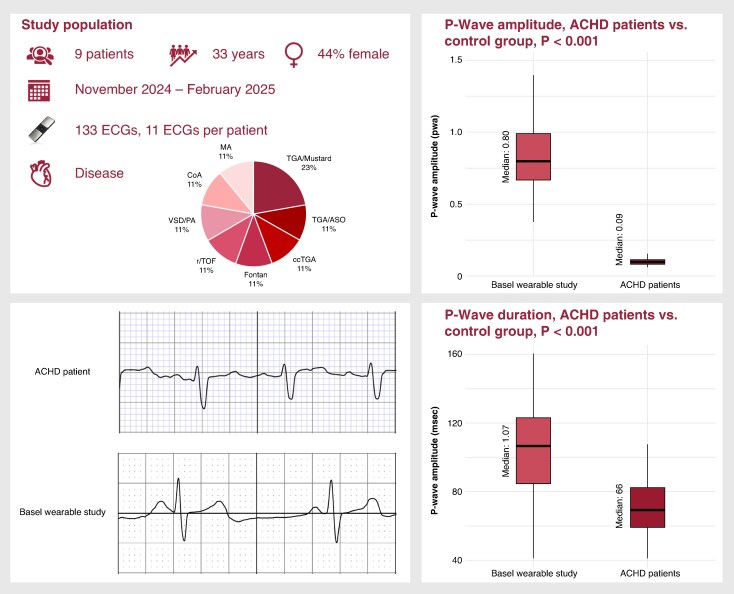
Right: Boxplots comparing P-wave amplitude (top) and P-wave duration (bottom) between ACHD patients (right) and a control cohort from the Basel Wearable Study (left). Bottom left: Representative ECG tracings from an ACHD patient (top) and from the Basel Wearable Study (bottom), highlighting differences in P-wave morphology. ACHD, adult congenital heart disease; ccTGA, congenital corrected transposition of the great arteries; CoA, repaired aortic coarctation; Fontan, Fontan circulation; MA, mitral atresia; r/TOF, repaired tetralogy of Fallot; TGA/ASO, transposition of the great arteries after arterial switch operation; TGA/Mustard, transposition of the great arteries after atrial switch; VSD/PA, ventricular septal defect with pulmonary atresia. Device image from AliveCor Inc., www.alivecor.com.

Our findings highlight several key challenges when applying wearable ECG monitoring in the ACHD population. First, adherence was suboptimal, with a median of only 11 ECGs submitted over 4 months, despite the inclusion of relatively young, digitally literate patients. Second, nearly half of the ECGs were classified as inconclusive, substantially higher than the 26% observed in the control population.^4^ Third, the significantly shorter and smaller P-waves observed in ACHD patients likely reflect atrial remodelling and conduction heterogeneity,^2^ which may not affect algorithms that rely primarily on RR irregularities, but could impair the diagnostic accuracy of manual review. These findings suggest that current smart devices may not perform reliably in ACHD patients. Careful patient selection, targeted patient engagement strategies, and tailored signal processing improvements may enhance future clinical utility in this subgroup. An opportunity to enhance patient adherence and improve disease awareness may lie in novel AI-based chat tools; however, a recent study showed limited medical appropriateness, which is particularly concerning in this complex patient cohort.^5^ Importantly, previous rhythm monitoring data using ILR confirmed that non-AF arrhythmias are frequently detected in ACHD patients,^3^ thereby reducing the yield of current smart devices for automated AF detection and underscoring the need for improved algorithms capable of reliably identifying (atypical) atrial flutter.^6^

The limitations are as follows: this was a single-centre pilot study with a small, heterogeneous patient cohort. No 12-lead ECG comparison was available as a reference standard, and no AF episodes occurred during the study, limiting evaluation of AF detection performance.

In conclusion, smart wearable ECG monitoring in ACHD patients was feasible but limited by high rates of inconclusive readings and reduced signal quality. Tailored algorithms and enhanced user engagement strategies are essential to optimize wearable ECG utility in this complex patient population.

## Data Availability

The data underlying this article will be shared on reasonable request to the corresponding author.
